# Variation in freshwater fish assemblages along a regional elevation gradient in the northern Andes, Colombia

**DOI:** 10.1002/ece3.1539

**Published:** 2015-06-04

**Authors:** Juan D Carvajal-Quintero, Federico Escobar, Fredy Alvarado, Francisco A Villa-Navarro, Úrsula Jaramillo-Villa, Javier A Maldonado-Ocampo

**Affiliations:** 1 Red de Ecoetología, Instituto de EcologíaA. C., Apartado Postal 63, Xalapa, Veracruz, 91000, México; 2 Lab. de Ictiología, Depto. de Biología, Facultad de Ciencias, Pontificia Universidad JaverianaCarrera 7 N° 43–82, Edf. 53 Lab. 108 B, Bogotá, Colombia; 3 Grupo de investigación en Sistemática Biológica SisBio, Escuela de Ciencias Biológicas, Universidad Pedagógica y Tecnológica de Colombia, Campus Universitario, Edificio Centro de Laboratorios primer pisoTunja, Boyacá, Colombia; 4 Grupo de Investigación en Zoología, Facultad de Ciencias, Universidad del TolimaIbagué, Colombia; 5 Instituto Alexander von HumboldtCalle 72 # 12 –65, piso 7, Bogotá, Colombia; 6 Lab. de Ictiología, Depto. de Biología, Facultad de Ciencias, Pontificia Universidad Javeriana, Unidad de Ecología y Sistemática (UNESIS)Carrera 7 N° 43–82 Edf. 53, Lab. 108 B, Bogotá, Colombia

**Keywords:** Diversity gradient, fish diversity, Neotropical mountains, functional groups, endemism, dendritic structure, headwaters

## Abstract

Studies on elevation diversity gradients have covered a large number of taxa and regions throughout the world; however, studies of freshwater fish are scarce and restricted to examining their changes along a specific gradient. These studies have reported a monotonic decrease in species richness with increasing elevation, but ignore the high taxonomic differentiation of each headwater assemblage that may generate high *β*-diversity among them. Here, we analyzed how fish assemblages vary with elevation among regional elevation bands, and how these changes are related to four environmental clines and to changes in the distribution, habitat use, and the morphology of fish species. Using a standardized field sampling technique, we assessed three different diversity and two structural assemblage measures across six regional elevation bands located in the northern Andes (Colombia). Each species was assigned to a functional group based on its body shape, habitat use, morphological, and/or behavioral adaptations. Additionally, at each sampling site, we measured four environmental variables. Our analyses showed: (1) After a monotonic decrease in species richness, we detected an increase in richness in the upper part of the gradient; (2) diversity patterns vary depending on the diversity measure used; (3) diversity patterns can be attributed to changes in species distribution and in the richness and proportions of functional groups along the regional elevation gradient; and (4) diversity patterns and changes in functional groups are highly correlated with variations in environmental variables, which also vary with elevation. These results suggest a novel pattern of variation in species richness with elevation: Species richness increases at the headwaters of the northern Andes owing to the cumulative number of endemic species there. This highlights the need for large-scale studies and has important implications for the aquatic conservation of the region.

## Introduction

For over 200 years, changes in the diversity and structure of the biological communities along elevation gradients have been of interest to ecologists, biogeographers, and conservationists. Elevation diversity gradients have been one of the most documented patterns in terms of spatial variation, and studies across them have covered a large number of biogeographic regions and terrestrial organisms (Sanders and Rahbek 2011). Two main species richness patterns with elevation have been observed: a unimodal (hump-shaped) pattern and a monotonic decrease with elevation, the first being the most frequently cited (Rahbek 2005).

Many factors have been proposed as underlying causes of elevation patterns, and they fall into four major themes: climatic, spatial, evolutionary, and biotic factors (Lomolino 2001; Grytnes and McCain 2007; Sanders and Rahbek 2011). Climatic factors determine how many species can stay alive at different locations and elevations. The variation and interaction between temperature, solar radiation, and precipitation across the gradient determine the local and regional patterns of productivity, which in turn limits population size and the total number of individuals. Furthermore, these variables impose physiological limits on species that define the minimum and maximum values within which a species can survive (Brown 2001; Hawkins et al. 2003). Spatial factors have been evaluated by examining the species–area relationship (SAR) and spatial constraint hypotheses (mid-domain effect). They predict how diversity patterns are restricted by area (Rahbek 1997) or spatial boundaries, respectively (Colwell et al. 2004). Evolutionary factors, such as rates of speciation, extinction, and colonization, vary across environmental gradients; thus, the number of species that could potentially occur at any point varies across the elevation gradient (Graham et al. 2014). Finally, different biotic processes have been proposed to explain patterns in species richness and include various levels of organization (e.g., population and community) and spatial scales (from microhabitat to landscape) (Grytnes and McCain 2007).

The importance of these factors in determining elevation gradients has been based on a large number of studies of terrestrial groups. In contrast to terrestrial ecosystems, there are few studies of fish diversity in freshwater environments along elevation gradients. The main recent reviews of the origin and maintenance of diversity in elevation gradients either made no mention of studies on freshwater fishes (see Sanders and Rahbek 2011), or it was the least represented taxonomic group (see Guo et al. 2013; Graham et al. 2014).

The elevation studies that have been performed on freshwater fish report a monotonic decrease in species richness with elevation (Fu et al. 2004; Jaramillo-Villa et al. 2010) and a hump-shaped pattern (Fu et al. 2004; Li et al. 2009). This pattern varies depending on how the community is defined, whether it is just the species of one taxonomic category (e.g., family), all the species in the assemblage (total species richness), or just endemic species. Studies of fish that use total species richness have reported a tendency for richness to decrease linearly as elevation increases (i.e., a low-elevation peak) (see Fu et al. 2004; Jaramillo-Villa et al. 2010). Similarly, at lower elevations, trophic composition is more diverse with detritivore, algivore, and piscivore species dominating the community; as elevation increases, the trophic structure becomes less diverse and insectivores dominate (Pouilly et al. 2006). Other factors such as the physicochemical characteristics of water have also been proposed as factors limiting fish distribution across elevations (Jaramillo-Villa et al. 2010).

Recent studies of other freshwater groups (macroinvertebrates, algae, and bacteria) that use total species richness have reported various patterns with elevation, some of them different from those reported for fish. For example, studies on macroinvertebrates report a *U*- and a hump-shaped response, while studies of bacteria and the epilithon (i.e., algae associated with rock surfaces) report an increase in species richness with elevation (see Wang et al. 2011; Lujan et al. 2013). Within a same taxonomic group, species richness patterns may vary with elevation depending on the scale of analysis. Nogués-Bravo et al. (2008) demonstrated that elevation diversity patterns vary as a function the extent or range of the elevation gradient that is sampled on the gradient being studied, tending to decrease linearly as the extent sampled decreases. This variation also depends on the location of any segment of the elevation gradient not sampled, particularly when it is a segment in the lower limit of the gradient that was excluded. Additionally, it is known that different patterns emerge with different scales of approach (local vs. regional) (Wiens 1989; Levin 1992); therefore, it is important not to assume that the patterns observed and the mechanisms that generate those patterns are similar at arbitrarily defined scales of analysis (Rahbek 2005). It is important to take into account local (specific gradients) and regional (watershed or regional gradients) approaches in spatial data analysis in ecology in order to detect the relationship of species with diversity (Jetz et al. 2005), an issue central to preventing the loss of biodiversity (Vinson and Hawkins 1998).

To the best of our knowledge, of the diversity studies that have been performed along elevation gradients in freshwater ecosystems, none have analyzed more than one elevation gradient at a time and as such do not describe the variation in diversity among regional elevation bands (i.e., these studies have only been performed on specific elevation gradients). Furthermore, some of these studies have omitted sampling one of the gradient’s segments; therefore, the elevation pattern described in these studies is, at the very least, incomplete and may lead to spurious conclusions or be biased.

To perform the first regional analysis of elevation diversity gradients in freshwater fish, we used 141 localities distributed between 250 and 2533 m a.s.l. in seven subregions in the Magdalena–Cauca River Basin. This river basin is located in the biogeographic region with the highest diversity of freshwater fish (the Neotropics), where more than 40% of the freshwater fish species are classified as endemic (Anderson and Maldonado-Ocampo 2011). Using this framework, we addressed the following questions: (1) How does freshwater fish diversity change along a regional elevation gradient? (2) How do species replacement (beta-diversity) and patterns of species co-occurrence vary with elevation? (3) How does the community structure of freshwater fish, particularly the functional groups, change as elevation increases? and (4) How do environmental variables influence species diversity and the relative proportions of the functional groups as elevation increases?

## Materials and Methods

### Study area

The northern Andes belong to a mountain system that extends from northern Peru (the Huancabamba Depression) to western Venezuela. In Colombia, the Andes represent an enormous mass of mountains that occupies approximately 30% of the country’s land area, and the Andean Cordillera diverges into three branches or ranges in Colombia separated by the valleys of the Magdalena and Cauca rivers. The lowlands of the Amazonian and Orinoquian regions are located at the eastern edge, and the Chocó biogeographic region is at the western edge. In the Colombian Andes, the rainfall patterns are bimodal, and regional variations in rainfall are associated with orographic effects produced by the steep topography. Winds from the Pacific and the Atlantic maintain constant humidity on both external slopes of the Cordilleras, while conditions are more variable on the inner flanks. Condensation is significant on the upper portions of the inner flanks, and the middle and lower portions of the valleys have a marked bimodal dry–wet pattern resulting from the rain shadow effect (Herzog et al. 2011).

These geographic and climate factors generate a diversity of freshwater systems with regional differences along elevation gradients. Colombian Andean streams are grouped into three categories based on their slope, substrate, and channel characteristics: (1) Lowland streams (≤1000 m a.s.l.) have gentle slopes (≤20°) and are characterized by wide and shallow channels, fine-grained substrata, and a high proportion of backwaters and pools; (2) mid-elevation streams (≥1000–1500 m a.s.l.) have a moderate slope (20° to 60°) and their channels are characterized as narrow and deep, with a substrate composed of isolated rocks of various sizes; and (3) highland streams (≥1500 m a.s.l.) have a steep slope (45° to 80°) and their channels are deep, the substrate is rocky, and the flow is torrential with no backwaters (Jaramillo-Villa et al. 2010).

### Sampling

**Figure 1:**
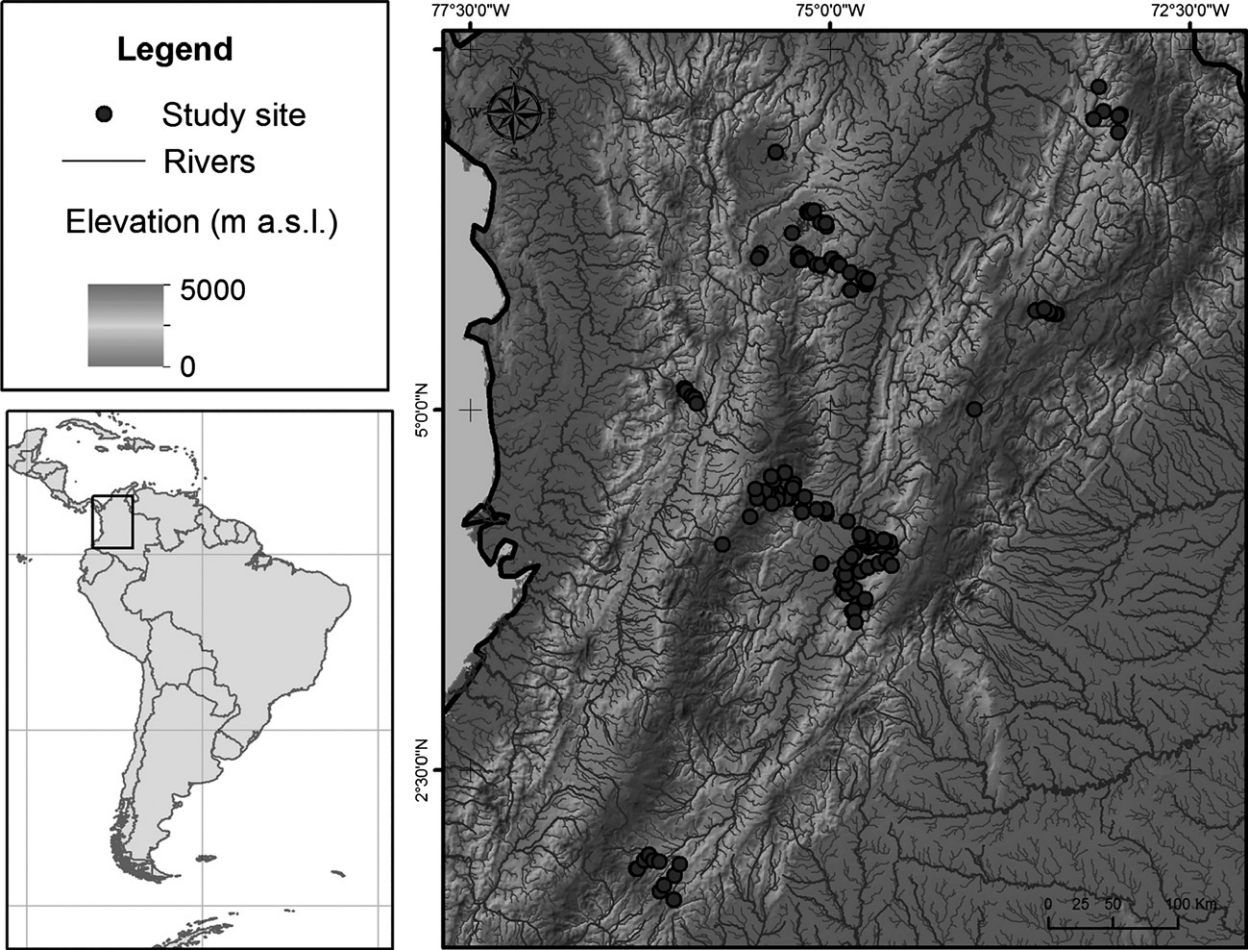
Location of the 141 sampling sites in the northern Andes, Colombia.

Between September 2003 and August 2005, we sampled 141 localities along seven gradients distributed throughout the Magdalena–Cauca River Basin, Colombian Andes (Fig.1, Table1). Sampling sites were located in first- to fourth-order streams (Strahler 1964) to reduce any longitudinal effect on the fish assemblages. This selection criterion follows Pouilly et al. (2006). The sampling sites were located between 256 and 2533 m a.s.l. (Table1) with a maximum depth and width of 2.5 and 30 m, respectively. At each site, we delimited a 100-m stretch where we carried out one sampling survey using electrofishing (340 V, 1–2 A, d.c.) along the section in an upstream direction. We also recorded the temperature (°C), conductivity (*μ*S cm^−1^), dissolved oxygen (mg L^−1^), and pH of the water using a portable multiparameter meter and obtained the elevation and geographic coordinates of each site from a global positioning system device.

**Table 1:** Distribution of the 141 sampling sites by region, elevation band (m a.s.l.), and stream category

Basin	Mountain Range	Subregion	Stream category					
			Lowland		Middle		High	
			≤500	1000	1250	1500	1750	≥2000
Magdalena	Central	Coello	4	5	4	3	3	10
Magdalena	Central	East of Antioquia	9	5	6	3	4	5
Magdalena	Central	Yalcones				7	1	3
Magdalena	Eastern	Prado	14	11	7	9	6	3
Magdalena	Eastern	Rasgón		3	1			2
Magdalena	Eastern	Iguaque						8
Cauca	Western	Tatamá		1	1	1		2
		Total	27	25	19	23	14	33

In the laboratory, we identified the fishes using taxonomic keys (Appendix S1) and by comparing them with identified material in the fish collection of the Alexander von Humboldt Institute (IAvH-P). We deposited voucher specimens in the fish collections of the IAvH-P, the Universidad Católica de Oriente (CP-UCO), and the Universidad del Tolima (CZUT-IC). The groups with taxonomic problems (e.g., Astroblepidae and Trichomycteridae) were revised by a specialist in Andean fishes, Javier A. Maldonado-Ocampo (author of the book *Peces de los Andes de Colombia*), who classified the undescribed species as morphotypes (using the morphological characters listed in Appendix S2). This classification allows us to make a conservative estimate of species richness for Andean fish groups with taxonomic problems, because it retains the same species as the molecular analysis, with the exception of cryptic species (Schaefer et al. 2011).

### Data analysis

To analyze the data, we defined regional elevation bands according to the physical features of the stream channels in the Colombian Andes (described above in Study area).

### Species richness reliability

The completeness of species richness was evaluated with the method proposed by Chao and Jost (2012) by contrasting species richness among elevation bands under the same sample coverage (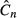), which is a measure of inventory completeness that gives the proportion of the total number of individuals in a community that belongs to each species represented in the sample (Chao and Jost 2012).

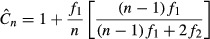


Sample coverage is based on the total number of individuals recorded (*n*), and on the number of rare species, singletons (*f*_1_) and doubletons (*f*_2_), that is, the species represented by one and two individuals, respectively (Chao and Jost 2012). When 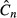 tends to zero, it indicates low completeness, while a value equal to 1 suggests that the sample has a high degree of completeness. The ecological relevance of 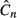 is that it does not violate the replication principle of diversity (Jost 2006; Chao and Jost 2012).

### Diversity patterns

The patterns of diversity were analyzed using Hill numbers (i.e., effective numbers of species, ^*q*^*D*). This analytical approach has been recognized as the most appropriate for evaluating diversity (Jost 2006, 2007; Chao et al. 2012). The formulas are detailed elsewhere (e.g., Jost 2006; Chao et al. 2012). We calculated the effective number of species ^0^*D* (species richness), ^1^*D* (exponential of Shannon’s entropy), and ^2^*D* (inverse Simpson concentration). ^0^*D* is not sensitive to species abundance and so gives disproportionate weight to rare species (Jost 2006).^1^*D* weights each species according to its abundance in the sample and therefore can be interpreted as the number of common (or typical) species in the community (Chao et al. 2012). ^2^*D* favors very abundant species and can be interpreted as the number of dominant species in the community (Chao et al. 2012).

To examine the relationship between elevation and the diversity of order *q* (^*q*^*D*), we used linear, quadratic, and cubic models to predict the best-fit model. The best explanatory model was selected based on the Akaike information criterion (AIC), where the model with the lowest AIC is the one that best fits the data. Likewise, using the best AIC fit, we analyzed the influence of the environmental variables (water temperature, DO, conductivity, and pH) on the different measures of diversity (^0^*D*, ^1^*D,* and ^2^*D*), identifying the best explanatory variables within each model. The models selected were checked, and when the variance was not constant and the errors were not normally distributed, we used a generalized linear model (GLM) assuming a Poisson distribution and using the log-link function (Crawley 2013).

### Beta-diversity

We used a multiplicative partition of diversity to obtain beta-diversity. For this, we calculated the rate of species turnover between adjacent elevation bands as (^q^*Dβ*–1)/(N–1), where N is the number of samples and ^q^*Dβ* is the *q* order beta-diversity, which is obtained from the quotient ^q^*Dγ*/^q^*Dα* (^q^*Dγ* is gamma diversity; ^q^*Dα* is alpha diversity – Jost 2007). We used the values of *q*0, 1, and 2. The rate of species turnover varies between 0 and 1 and can be expressed as a percentage. It is zero when there is no species turnover and 100% when the species composition at each elevation band is completely different (Jost 2007). Given that the rate of species turnover does not reveal whether the replacement is the result of a loss or gain of species, we calculated gains and losses in the number of species for each comparison. Additionally, we calculated total *β* (*β*_t_) for the three orders of *q*, to determine whether fish assemblages differ in structure or composition across the elevation gradient (using EstimateS v.9.1; Colwell 2005). Based on the number of different communities that we found in the *β*_t_ analysis, we performed a test of homogeneity of dispersion (PERMDISP; Anderson 2006) to corroborate the differences in assemblage composition among these assemblages.

### Community structure

To evaluate any changes in the fish community structure along a regional elevation gradient, we assessed the dominance and rarity of species. Dominance was evaluated by plotting the species rank abundance for each elevation band. As the slope of the abundance distribution decreases and the range of this slope increases with respect to the *x*-axis, dominance within the assemblage decreases. This method is widely used to compare differences in structure among disparate communities (i.e., communities with different sizes and with few or no species in common) (McGill et al. 2007; Magurran and McGill 2011). Species rarity was assessed for each elevation band by graphing the relative frequency at which a certain number of shared species occurs with respect to the total number of sites sampled. This measure also illustrates how vertical and horizontal (i.e., among subregions) distributions vary across the regional elevation gradient.

Using a presence–absence matrix, we tested the existence of nonrandom patterns of species co-occurrence using the ECOSIMR package version 1.0 (Gotelli and Ellison 2013). We generated 5000 Monte Carlo randomized matrices. Each locality had the same number of species as in the real data, and each species in the null communities had the same number of occurrences as in the original data. We calculated the C-score co-occurrence index (Stone and Roberts 1990) to statistically compare the patterns in the randomized communities with those in the real data matrix. We ran the co-occurrence test twice: once under the assumption that all sites have an equal probability of being chosen, and the second under the assumption that the probability of choosing a locality is proportional to its elevation (i.e., that the occurrence of species is not randomly distributed among localities and depends on elevation).

### Functional groups

We assigned each species to a functional group according to Maldonado-Ocampo et al. (2005) and Jaramillo-Villa et al. (2010). This classification is based on body shape, habitat use, morphological, and/or behavioral adaptations, and it separates species into five groups: torrent, pool, pelagic, rheophilic, and nontorrent benthic (Appendix S3). Using linear models, we evaluated the relationship between elevation and the abundance and richness of each functional group. We also analyzed the effect of the environmental variables on the abundance and species richness of functional groups.

## Results

**Figure 2:**
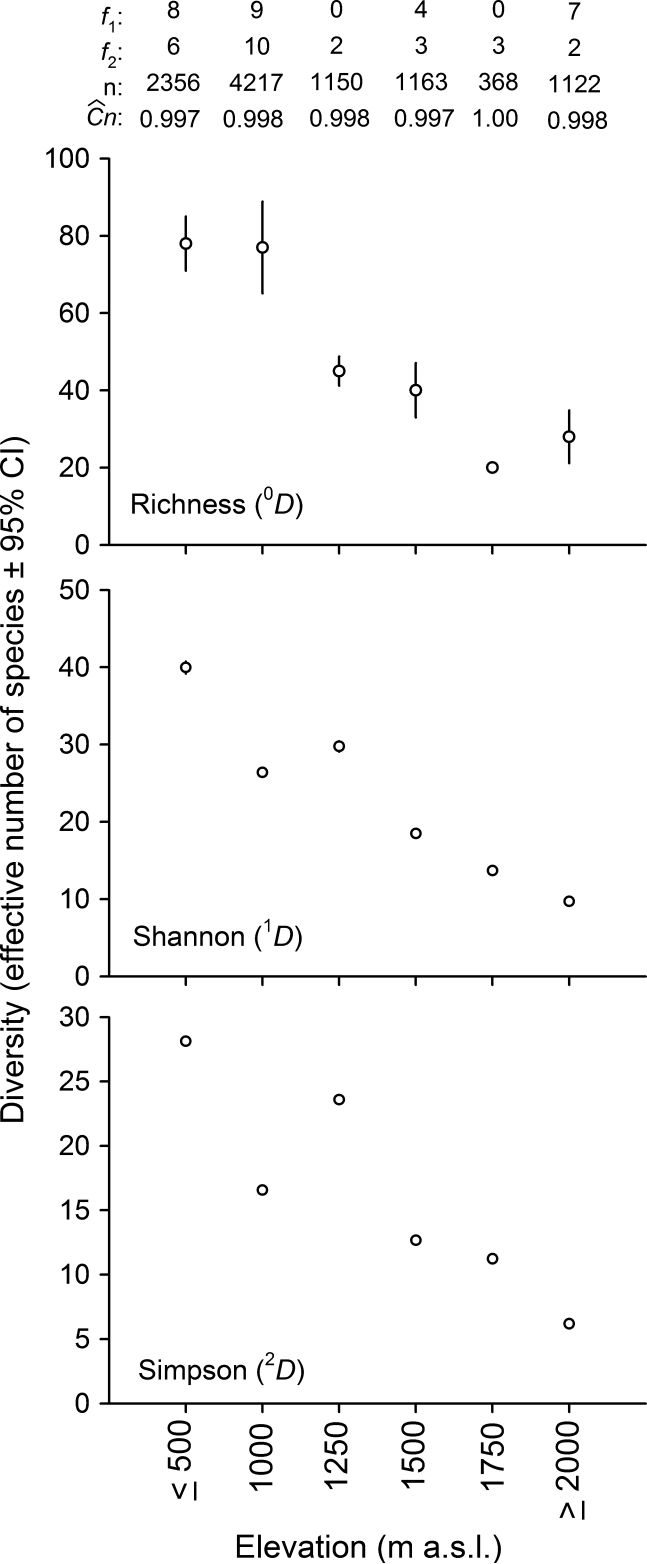
Diversity values of order *q* (^*q*^D) for each regional elevation band, sample coverage, and abundance for each elevation band. *f*_1_: singletons, *f*_2_: doubletons, *n*: total number of individuals, 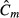: sample coverage. Vertical bars represent the CI for each value of ^*q*^D in each elevation band. The CI of ^*1*^*D* and ^*2*^*D* is not apparent because of their low values.

We captured a total of 10376 individuals belonging to 137 species, 51 genera, 23 families, and seven orders (see Appendix S4). The most species-rich families were Astroblepidae (1 genus and 31 species), Characidae (11 genera and 30 species), Loricariidae (12 genera and 20 species), and Trichomycteridae (1 genus and 15 species). The number of localities sampled per elevation band varied between 14 localities in the 1750 m a.s.l. band and 33 localities in the ≥2000 m a.s.l. band (Table1); however, species richness did not depend on the number of sites sampled (*R*^2^ = 0.09). Overall, a large proportion of the species present in each elevation band was recorded (coverage deficits <1%; Fig.2); thus, comparisons of species diversity and composition were possible.

Although the three measures of diversity (^0^*D*, ^1^*D*, ^2^*D*) had a statistical tendency to decrease linearly as elevation increased (^0^*D*: *R*^2^ = 0.80, df = 4, *P* = 0.009, AIC = 49.15; ^1^*D*: *R*^2^ = 0.80, df = 4, *P* = 0.009, AIC = 34.28; ^2^*D*: *R*^2^ = 0.77, df = 4, *P* = 0.012, AIC = 36.79), there were variations depending on which measure of diversity was used. The highest species richness (^0^*D*) was found at lower elevations ≤500 and 1000 m a.s.l.; species richness decreased with increasing elevation up to the 1750 m a.s.l. band; above 2000 m a.s.l., species richness increased (Fig.2). However, diversity patterns of common (^1^*D*) and dominant species (^2^*D*) decreased with increasing elevation to 2000 m a.s.l., but there was a marked increase at 1250 m a.s.l. (Fig.2). An a posteriori comparison of the species richness pattern without the unresolved taxonomic species (i.e., morphospecies) shows that the general trend is unchanged, but the magnitude of the increase in species richness in the upper portion of the gradient is smaller (Appendix S5).

**Figure 3:**
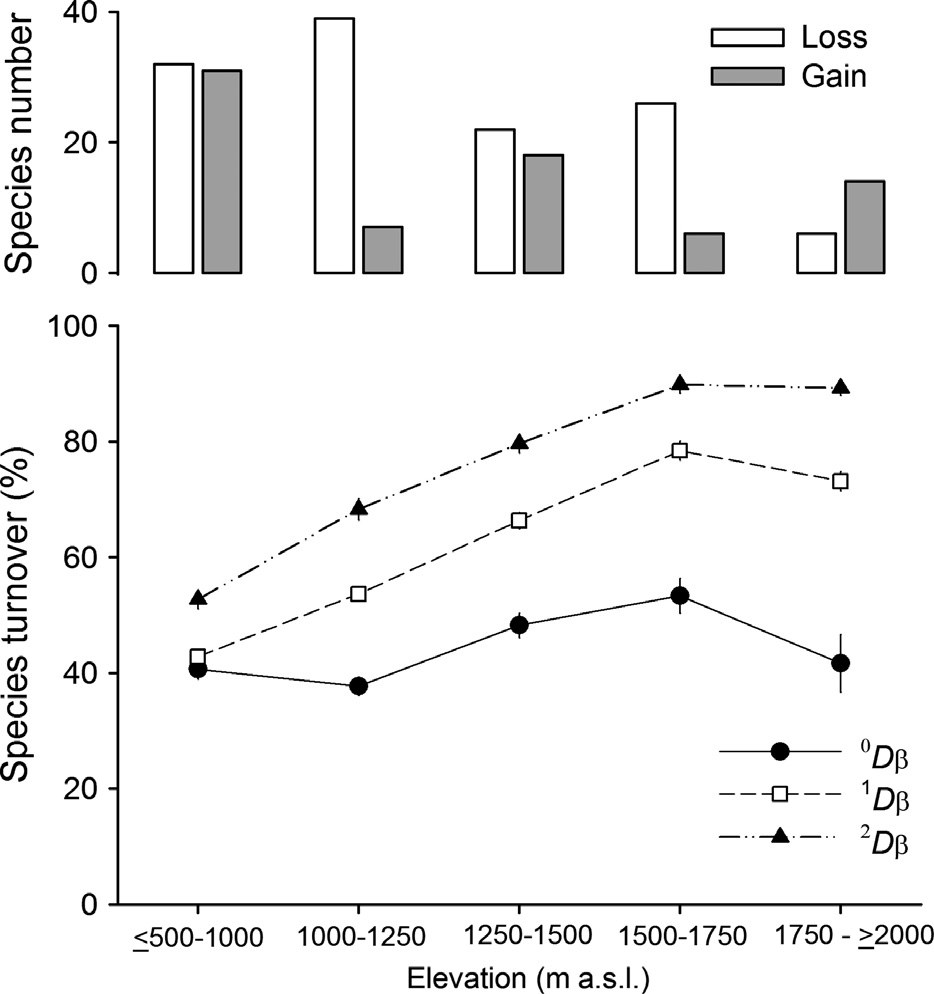
Above: gain and loss in the number of species between adjacent elevation bands. Below: *β*-diversity values of order *q* (^*q*^D), for adjacent elevation bands. Vertical lines represent 95% CI.

The species turnover analysis revealed an increase in the distinctiveness of the fish fauna with increasing elevation and a general decrease in turnover for the streams above 2000 m a.s.l. Although species turnover patterns also changed in magnitude depending on the ^q^*Dβ* used, we observed that species replacement was highest in the highland streams and that turnover across the gradient is mainly due to changes in typical (^1^*D*) and dominant (^2^*D*) species (Fig.3). Species loss was higher than gain at all elevations except for the streams above 2000 m a.s.l. There were two marked occurrences of species loss (between 1000–1250 m a.s.l. and 1500–1750 m a.s.l.) followed by noticeable species gains (at 1250 m a.s.l. and 2000 m a.s.l.). Total *β*_t_ diversities also indicated high species turnover, showing that along the elevation gradient, there are two communities with different compositional and structural characteristics (^0^*Dβ*_t_ = 2.85, ^1^*Dβ*_t_ = 2.97, ^2^D*β*_t_ = 3.24). The test of homogeneity of dispersion corroborates the differences in assemblage composition between lowland streams and highland assemblages (*t *= 3.3086, *P* = 0.0013).

**Figure 4:**
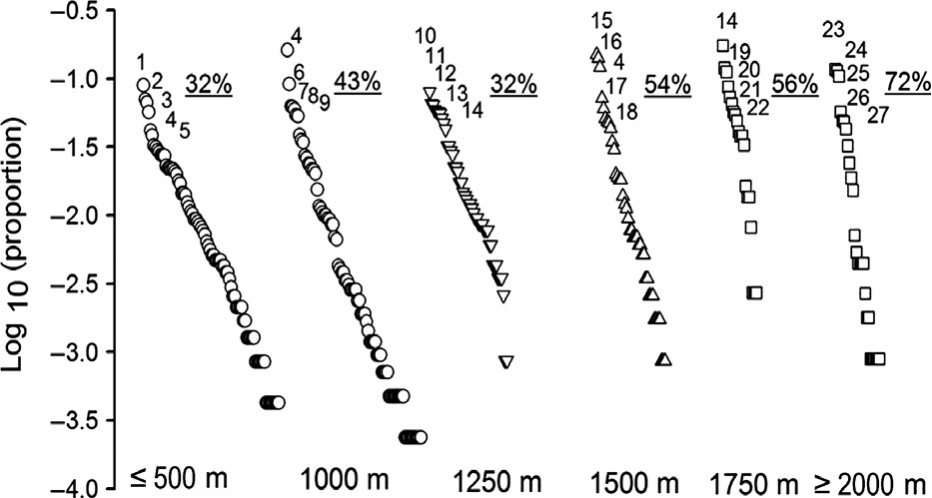
Species rank abundance for each elevation band. The cumulative percentage of abundance for the five most abundant species in each elevation band is presented. Note that the composition of the most abundant species in each elevation band is different. *Lasiancistrus caucanus* (1), *Gephyrocharax melanocheir* (2), *Microgenys minuta* (3), *Trichomycterus banneaui* (4), *Argopleura magdalenensis* (5),*Chaetostoma thomsoni* (6), *Trichomycterus* sp. 5(7), *Creagrutus magdalenae* (8), *Chaetostoma leucomelas* (9), *Bryconamericus* sp. 3(10), *Astroblepus homodon* (11), *Hemibrycon tolimae* (12), *Trichomycterus striatus* (13), *Astroblepus longifilis* (14), *Hemibrycon boquiae* (15), *Creagrutus brevipinnis* (16) *Bryconamericus huilae* (17), *Astroblepus* sp. 16 (18), *Brycon henni* (19), *Trichomycterus caliensis* (20), *Astroblepus chotae* (21), *Astroblepus* sp. 11 (22), *Astroblepus chapmani* (23), *Astroblepus cirratus* (24), *Astroblepus micrescens* (25), *Geophagus steindachneri* (26), and *Dolichancistrus carnegiei* (27).

**Figure 5:**
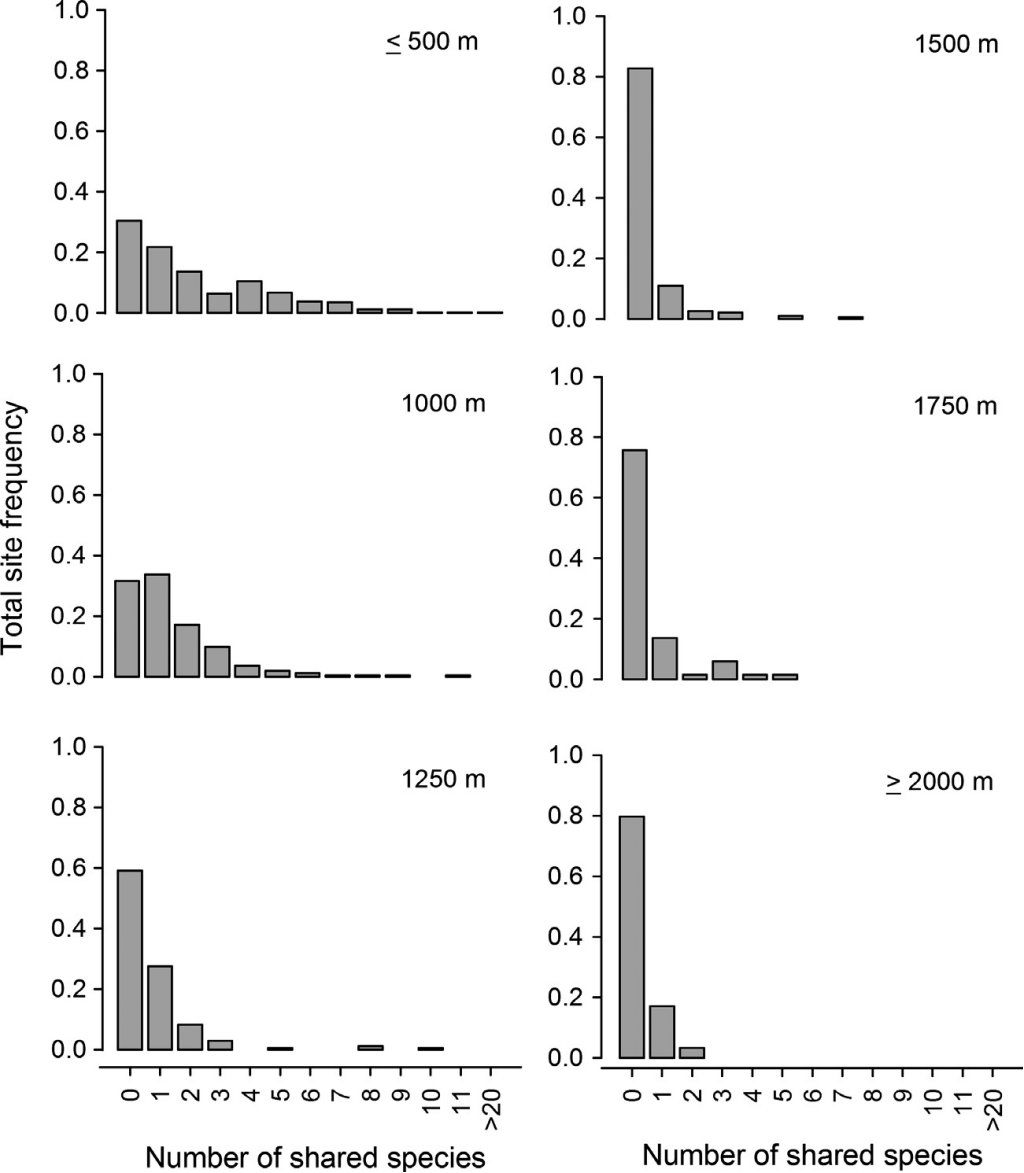
Relative frequency at which a certain number of shared species occur with respect to the total number of sites in each elevation band.

The fish assemblage structure changed across our regional elevation gradient (Fig.4). As elevation increases, the general pattern observed was an increase in the dominance of a few species that remain in the assemblage (*Astroblepus* spp. and other endemic species, Appendix 4). Moreover, the species that dominate the assemblage are different in each elevation band (Fig.4). Species rarity and distribution also varied with elevation. In lowland streams (below 1000 m a.s.l.), species tended to be present at a greater number of sites and the frequency of unique species was low (about 30% of species occupy a single locality); with increasing elevation, the number of unique species was higher and the species tended to occupy few sites. Almost 80% of the species were specific to a single site in the highland streams (Fig.5).

The average C-score for all pairwise matrix values among species was 25.1, and none of the simulated matrices had a C-score greater than the observed (mean simulated indices 24.7). Thus, fish species co-occurrence was much lower in the observed matrix than expected by chance (*P* < 0.0001), suggesting that the fish assemblage was spatially structured. When each site was proportionally weighted according to its elevation (so that sites at a lower elevation would have a greater chance of receiving species), simulated matrices also had a significantly lower C-score than observed (22.57, *P* < 0.0001), showing that elevation was a factor that structured the fish assemblage. Temperature was the only environmental variable that explained the variation in fish species diversity along the elevation gradient (^0^*D*: *R*^2^ = 0.75, df = 4, *P* = 0.035, AIC: 36.52; ^1^*D*: *R*^2^ = 0.95, df = 4, *P* = 0.0003, AIC: 10.47; ^2^*D*: *R*^2^ = 0.85, df = 4, *P* = 0.003, AIC: 32.4).

### Functional groups

**Figure 6:**
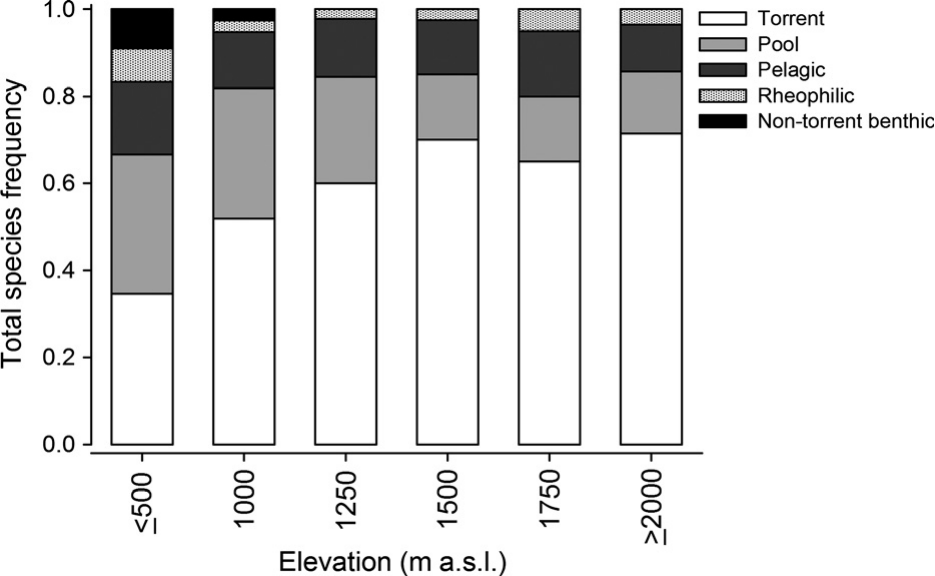
Total species frequency of each functional group in each elevation band.

The proportion of species in each functional group depended on elevation (*χ*^2^ = 32.75, df = 20, *P* < 0.04, Fig.6). The proportion of torrent species increased significantly toward the headwaters (AIC = 30.20, df = 4, *P* < 0.0001), reaching its highest value at the highest elevations. The proportion of pool species was the inverse of that of the torrent species, with the highest values recorded for the lowland streams (AIC = 13.8, df = 4, *P* = 0.003). Nontorrent benthic species were restricted to the lowland streams (AIC = 11.5, df = 4, *P* = 0.005), and pelagic and rheophilic species were relatively constant across the regional elevation gradient we studied (AIC = 25.1, df = 4, *P* = 0.449; AIC = 19.3, df = 4, *P* = 0. 234; respectively).

The richness of torrent species was not affected by elevation (*R*^2^ = 0.20, df = 4, *P* = 0.206); they were present in notable numbers throughout the gradient. Richness for the other functional groups decreased with increasing elevation (pool: *R*^2^ = 0.84, df = 4, *P* = 0.006; pelagic: *R*^2^ = 0.93, df = 4, *P* = 0.001; rheophilic: AIC = 19.1, df = 4, *P* = 0.012; nontorrent benthic: AIC = 12.35, df = 4, *P* = 0.001).

Of the four physicochemical parameters measured for the water (temperature, DO, conductivity, and pH), temperature and DO better explained the variation in species richness with elevation in functional groups. For pelagic, rheophilic, and nontorrent benthic fish, species richness was strongly correlated with temperature and DO (pelagic DO: *R*^2^ = 0.84, df = 4, *P* = 0.005, temperature: *R*^2^ = 0.83, df = 4, *P* = 0.007; rheophilic temperature: AIC = 19.013, df = 4, *P* = 0.0111; DO: AIC = 19.013, df = 4, *P* = 0.0111, nontorrent benthic temperature: AIC = 12.28, df = 4, *P* = 0.001, DO: AIC = 10.89, df = 4, *P* = 0.019). For torrent species, temperature was the only variable that was correlated with species richness (*R*^2^ = 0.78, df = 4, *P* = 0.012); for pool species, there was a correlation with DO (*R*^2^ = 0.82, df = 4, *P* = 0.007).

## Discussion

Our results suggest a novel pattern of variation in species richness with elevation: There was drop in species richness from the lower to middle elevations along our regional gradient, but the number of species tended to increase above 2000 m a.s.l (Fig.2). Several studies have claimed a high taxonomic richness in headwater assemblages for different freshwater groups (see Clarke et al. 2008; Besemer et al. 2013; Göthe et al. 2014). When river systems are viewed at a regional scale (watershed scale), they are branching systems in which the headwaters and tributaries are the major component of the network and may account for more than three-quarters of the length of all stream channels within a watershed (Hansen 2001). Headwater assemblages have a high degree of taxonomic differentiation with respect to lowland assemblages (Alofs et al. 2014) and those of other headwaters (Clarke et al. 2008; Besemer et al. 2013; Göthe et al. 2014) owing to their isolation, the high levels of endemism (Mayden 1988; Alofs et al. 2014), and singular evolutionary history (Hoagstrom et al. 2014). Thus, considering the spatial structure of headwater systems and the spatial diversity characteristics of the assemblages that inhabit these environments, headwater assemblages may have low *α*-diversity in individual headwater streams, but high *β*-diversity at the headwaters generates high *γ*-diversity (Clarke et al. 2008). This pattern also emerges in the genetic structure of some groups of headwater stream macroinvertebrates (Hughes 2007; Finn et al. 2011). Previous studies (along individual gradients) report a monotonic decline in species richness with increasing elevation (Fu et al. 2004; Jaramillo-Villa et al. 2010; Lujan et al. 2013), because they ignore the average contribution of local streams to *γ*-diversity.

We observed that the diversity of common (^1^*D*) and dominant (^2^*D*) species decreased with increasing elevation, with marked humps at 1250 m a.s.l. According to Lomolino (2001), this suggests that there is contact and mixing of faunas with different climate and habitat tolerances (from the lower and upper streams), probably as a consequence of the different historical biogeography of the lineages. This contact marks a change in the assemblage structure along the elevation gradient (i.e., dominant species), reflected in the total *β*-diversity values and represented by two fish fauna: a lowland fish fauna and a highland fish fauna with the highest dominance values.

*β*-Diversity analyses between adjacent pairs of elevation bands also support the presence of different fish faunas, revealing an increase in the distinctiveness and higher species losses than gains toward the headwaters, with the exception of the streams above 2000 m a.s.l. where distinctiveness decreases and species gain is higher than loss, suggesting a different fauna in headwater streams. Kraft et al. (2011) suggested that these changes in *β*-diversity across large-scale diversity gradients are related to the variation in the species pool, set by the variation in biogeographic or regional processes. In freshwater fish, the natural longitudinal fragmentation of river networks, caused by natural waterfalls and rapids, has been proposed as a biogeographic factor that shapes ecological communities across large-scale diversity gradients, on the basis that they could act as ecological barriers limiting fish dispersal processes and isolating populations (Oberdorff et al. 1999; Dias et al. 2013). In addition, the dendritic spatial structure of river networks (which causes horizontal fragmentation, as mentioned above) and the low dispersal ability of the highland biota have also been identified as potential mechanisms that generate high beta-diversity in highland streams (Clarke et al. 2008; Finn et al. 2011). They promote high rates of genetic differentiation within the species (Hughes 2007) and low rates of immigration of new species into local assemblages from the regional species pool (Fu et al. 2004; Clarke et al. 2008), resulting in a highly differentiated biota as isolation increases toward the headwaters.

The increase in isolation is reflected in our species rarity and distribution results, which suggest that as elevation increases, species tend to (1) reduce their elevation distribution; (2) restrict their horizontal distribution to a single stream channel; and (3) result in the co-occurrence of fewer species. As mentioned above, the physiographic conditions of rivers are more complex and fragmented toward the headwaters, promoting fish assemblage isolation (Campbell Grant et al. 2007) and high turnover among fish assemblages. High levels of neoendemism (endemism originating from speciation within a single drainage basin, sensu Tedesco et al. 2012) are promoted by isolation (Oberdorff et al. 1999; Fu et al. 2004; Tedesco et al. 2012). In fact, the northern Andes have the highest levels of fish endemism in their headwater streams (Maldonado-Ocampo et al. 2005; Anderson and Maldonado-Ocampo 2011). This high level of endemism in the highland streams suggests that the increased species richness in this region results from the cumulative number of endemic species in the highland streams. The peak in species richness at upper elevations due to high levels of endemism has already been proposed by Lomolino (2001), who stated that species densities should peak in these zones because they may represent hotspots of speciation and endemism.

Changes in community attributes with elevation seem to be related to changes in habitat use and the morphological traits of species (i.e., changes in the composition and structure of functional groups). Along an elevation gradient, water bodies change from very swift, turbulent, cold, and highly oxygenated at the highest elevations to less turbulent and oxygenated, more turbid and warmer in the lowest reaches (Jacobsen 2008). In terms of habitat, headwater streams are mostly composed of rapids and waterfalls and these change toward the lowlands, where streams have a greater habitat diversity and are composed of riffles, runs, and different kinds of pools (Scatena and Gupta 2012). Changes in the composition of functional groups along the elevation gradient parallel the changes in habitat characteristics with increasing elevation. At lower elevations where there is a greater diversity of habitats, there are a larger number of functional groups, and with increasing elevation, habitat and functional group diversity decreases (Fig.6). Additionally, torrential species (i.e., those that dominate the upper portion of the gradient) are adapted to the harsh environmental conditions of Andean headwater streams. They have developed different morphological and behavioral adaptations, including body shapes that reduce water resistance, suction-cup-like appendages for clinging to bedrock surfaces and climbing waterfalls, and the ability to hide under large boulders or occupy shallow waters in channel-margin habitats during floods and drought (Maldonado-Ocampo et al. 2005; Scatena and Gupta 2012; Carvajal-Quintero et al. 2015).

Water temperature and dissolved oxygen (DO) are related to the observed changes in species diversity. These variables have been proposed as responsible for the changes in the freshwater assemblages across elevation gradients (Allen 1995; Irz et al. 2007; Jaramillo-Villa et al. 2010; this study). Temperature and oxygen availability affect directly organisms’ performance, limiting growth, metabolism, breeding, development and affecting the behavior of fishes (Buisson et al. 2008; Verberk et al. 2011), this in turn limit species’ distribution and shape noticeable diversity gradients in these organisms. Additionally, it is known that aquatic organisms are more sensitive to temperature changes than terrestrial organisms are, given that the large thermal mass of water buffers the changes in land temperature (Clarke and Johnston 1999).

Although in this study we show how some water variables (temperature and DO) are related to changes in the fish community assemblages along a regional elevation gradient, it is necessary to determine the effect of other environmental variables and biotic interactions on these patterns. Future studies should take into account the high topographic variability of the watersheds located in complex mountain systems such as the northern Andes. Although we grouped sampling points according to the general physiographic characteristics associated with elevation in northern Andean streams, this does not mean every locality sampled that is located in a given elevation range belongs to a specific river category in any subregion of the Andes.

This study reveals the importance of large-scale studies, which can elucidate diversity patterns different from those revealed by smaller-scale studies. The novel pattern of fish species richness that we report highlights the river headwaters as environments with great regional species richness and containing a high proportion of endemic fishes that are headwater specialists (Clarke et al. 2008; Finn et al. 2011; Besemer et al. 2013; Alofs et al. 2014). Thus, it is important for new conservation strategies to change the linear perception of river systems and incorporate the effects of their branching structure. This will make it possible to conserve the large number of neoendemic species that are restricted to isolated sections of basins such as tributaries and the headwaters. Conserving endemic species is an important strategy because it allows us to protect a greater number of species than would occur by chance (Lamoreux et al. 2006).

Finally, we wish to emphasize the value of improving our taxonomic knowledge as it makes the accurate evaluation of diversity patterns possible. Tropical freshwater systems, including those of the Neotropics, are poorly understood. The large number of unknown species along with a historical sampling bias toward more accessible areas has limited the accurate evaluation of diversity and biogeographical patterns (Albert et al., 2011). This became evident on comparing the species richness pattern between a resolved and unresolved taxonomic assemblage (i.e., assemblages with and with no morphospecies, respectively); patterns with different magnitudes of change were revealed. The current rates of species description suggest that the species richness and number of undescribed species in the Neotropics have been underestimated (Reis, 2013). The Magdalena–Cauca River Basin is an example of this situation; of the total number of new freshwater fish species, descriptions in Colombia in the last 10 years, almost 50% (around 55 species), are from this river basin, and almost all of these new species are from tributaries that drain from the Andean highlands to the main channel in the lowlands. More descriptions are in progress as a result of new collecting efforts in this river basin, and revisionary studies are underway on groups for which there is taxonomic conflict such as the Trichomicterid, Astroblepid, and Loricariid catfish (Maldonado-Ocampo, per. obs.). These taxonomic and collecting efforts will contribute to a better understanding of the new regional diversity patterns with elevation presented here and will provide information to the stakeholders, so they can develop appropriate conservation strategies for the most important river basin in the northwest of South America.

## Supporting Information

**Appendix S1.** List of taxonomic keys used to identify the freshwater fish specimens from a regional elevation gradient in the Northern Andes, Colombia. **Appendix S2.** Morphological characters used to delineate morphospecies within families with taxonomic conflicts: color pattern (A), kind of adipose fin (B), origin of pelvic fins (C), kind of teeth in the outer series of the premaxillary and mandibular ramus (D), number of teeth in the outer series of the premaxillary and mandibular ramus (E), length of the pectoral fins (F), number of teeth in the inner row of the premaxilla (G), tooth pattern in the inner row of the premaxilla (H), number of scales on the lateral line (I), number of lateral scutes (J), presence/absence and kind of cheek odontodes (K), presence/absence of supraoccipital dermal keel (L), number of dorsal fin rays (M), number of anal fin rays (*N*), and number of pectoral fin rays (O). **Appendix S3.** Criteria used to define the functional groups of the freshwater fish inhabiting a regional elevation gradient in the Northern Andes, Colombia. **Appendix S4.** Species and abundance of fishes collected at each elevation band and functional group (T, torrent; N-t b, nontorrent benthic; Po, pool; Pe, pelagic; R, rheophilic) along a regional elevation gradient of the Northern Andes, Colombia. **Appendix S5.** Diversity values of order zero (^0^*D*, species richness) for each regional elevation band. Assemblages with morphospecies are black circles and those with no morphospecies are white circles.
