# Supplementary material for: Variation in freshwater fish assemblages along a regional elevation gradient in the northern Andes, Colombia
Source: Ecol Evol. 2015 Jun 4;5(13):2608–20. doi: 10.1002/ece3.1539 (PMC4523357; doi:10.1002/ece3.1539)
Supplement: Supplementary file 1 [file ece30005-2608-sd1.docx]

**Appendix S1.** List of taxonomic keys used to identify the freshwater fish specimens from a regional elevation gradient in the Northern Andes, Colombia.

Armbruster, J. (2005) The Loricariid catfish genus *Lasiancistrus* (Siluriformes) with descriptions of two new species. *Neotropical Ichthyology*, **3**, 549–569.

Dahl, G. (1971) Los peces del norte de Colombia. Instituto de Desarrollo de los Recursos Naturales Renovables (INDERENA), Talleres de Litografía Arco.

Eigenmann, C.H. (1918) The Pygidiidae, a family of South American catfishes. *Memoirs of the Carnegie Museum*, **7**, 259–399.

Géry, J. (1977) Characoids of the World. T.F.H. Publications Ltd.

Maldonado–Ocampo, J.A., Ortega-Lara, A., Usma, J.S., Galvis, V.G., Villa-Navarro, F.A., Vásquez, G.L., Prada-Pedreros, S. & Ardila, R.C. (2005) Peces de los Andes de Colombia. Instituto Alexander von Humboldt, ARFO Editores.

Román-Valencia, C. (2003) Sistemática de las especies colombianas de *Bryconamericus* (Characiformes, Characidae). *Dahlia*, **6**, 17–58.

Vari, R. & Harold, A. (2001) Phylogenetic study of the Neotropical fish genera *Creagrutus* Günther and *Piabina* Reinhardt (Teleostei: Ostariophysi: Characiformes), with a revision of the Cis-Andean species. *Smithsonian Contributions to Zoology*, **613**, 1–131.

Vari, R., Ferraris, C. & Pinna, M. (2005) The Neotropical whale catfish (Siluriformes: Cetopsidae: Cetopsinae) a revisionary study. *Neotropical Ichthyology*, **3**, 127–238.

**Appendix S2.**  Morphological characters used to delineate morphospecies within families with taxonomic conflicts: color pattern (A), kind of adipose fin (B), origin of pelvic fins (C), kind of teeth in the outer series of the premaxillary and mandibular ramus (D), number of teeth in the outer series of the premaxillary and mandibular ramus (E), length of the pectoral fins (F), number of teeth in the inner row of the premaxilla (G), tooth pattern in the inner row of the premaxilla (H), number of scales on the lateral line (I), number of lateral scutes (J), presence/absence and kind of cheek odontodes (K), presence/absence of supraoccipital dermal keel (L), number of dorsal fin rays (M), number of anal fin rays (N), and number of pectoral fin rays (O).

| Character | Family | | | | | | | |
| --- | --- | --- | --- | --- | --- | --- | --- | --- |
|  |  |  |  |  |  |  |  |  |
|  | Parondontidae | Crenuchidae | Characidae | Trichimycteridae | Astroblepidae | Loricariidae | Heptapteridae | Rivulidae |
|  |  |  |  |  |  |  |  |  |
| A | X | X |  | X | X |  |  |  |
| B |  |  |  | X | X | X | X | X |
| C |  |  |  |  | X | X | X |  |
| D |  |  |  |  | X |  |  |  |
| E |  |  |  |  | X | X |  |  |
| F | X | X | X |  |  |  |  |  |
| G |  |  | X |  |  |  |  |  |
| H |  |  | X |  |  |  |  |  |
| I | X |  | X |  |  |  |  |  |
| J |  |  |  |  |  | X |  |  |
| K |  |  |  | X |  | X |  |  |
| L |  |  |  |  |  | X |  |  |
| M | X | X | X |  |  |  |  | X |
| N | X | X | X |  |  |  |  | X |
| O | X | X | X |  |  |  |  | X |

**Appendix 3.** Criteria used to define the functional groups of the freshwater fish inhabiting a regional elevation gradient in the Northern Andes, Colombia.

| Functional Group | Body size | Body Shape | Morphological and/or behavioral adaptations | Habitat |
| --- | --- | --- | --- | --- |
| Torrent | Small to medium | Dorso-ventrally compressed | Reduced swim bladders that permit the fish to adhere to rocks and move upstream in fast currents. Sucker mouths, odontodes or other appendages that allow them to anchor to the substrate. | Rapids and torrent waters |
| Non-torrent benthic | Medium to large | Dorso-ventrally compressed | Suckermouths, well-developed barbels and reduced swim bladders that permit them to live attached to the substrate. Some species have different kinds of migrations | Riverbeds |
| Pool | Small to medium | Laterally compressed | Well-developed swim bladders and shoals live | Pools |
| Pelagic | Wide range of body sizes | Fusiform bodies with hydrodynamic shapes | Do not migrate | Water column of fast currents and pools |
| Rheophilic | Medium to large | Fusiform or dorso-ventrally compressed bodies with hydrodynamic shapes | Have medium and long migrations. | Water column of fast currents and pools |

**Appendix 4.** Species and abundance of fishes collected at each elevation band and functional group (T, torrent; N-t b, non-torrent benthic; Po, pool; Pe, pelagic; R, rheophilic) along a regional elevation gradient of the Northern Andes, Colombia. The groups with taxonomic problems were reviewed by Javier A. Maldonado-Ocampo, a specialist in Andean fish and author of the book "Peces de los Andes de Colombia" (see Maldonado-Ocampo et al. 2005).

| Species | Elevation band (m a.s.l.) | | | | | |  | Functional groups |
| --- | --- | --- | --- | --- | --- | --- | --- | --- |
|  | < 500 | 1000 | 1250 | 1500 | 1750 | > 2000 | Total |  |
| Order Myliobatiformes |  |  |  |  |  |  |  |  |
| Family Potamotrygonidae |  |  |  |  |  |  |  |  |
| *Potamotrygon magdalenae* | 5 | 0 | 0 | 0 | 0 | 0 | 5 | N-t b |
|  |  |  |  |  |  |  |  |  |
| Order Characiformes |  |  |  |  |  |  |  |  |
| Family Parodontidae |  |  |  |  |  |  |  |  |
| *Parodon caliensis* | 0 | 2 | 0 | 0 | 0 | 0 | 2 | T |
| *Parodon magdalenensis* | 47 | 10 | 0 | 0 | 0 | 0 | 57 | T |
| *Parodon* sp. | 0 | 2 | 0 | 0 | 0 | 0 | 2 | T |
| *Saccodon dariensis* | 0 | 7 | 7 | 2 | 0 | 0 | 16 | T |
| Family Curimatidae |  |  |  |  |  |  |  |  |
| *Cyphocharax magdalenae* | 1 | 0 | 0 | 0 | 0 | 0 | 1 | R |
| Family Prochilodontidae |  |  |  |  |  |  |  |  |
| *Prochilodus magdalenae* | 2 | 0 | 0 | 0 | 0 | 0 | 2 | R |
| Family Crenuchidae |  |  |  |  |  |  |  |  |
| *Characidium fasciatum* | 0 | 0 | 0 | 11 | 0 | 0 | 11 | T |
| *Characidium phoxocephalum* | 5 | 16 | 9 | 0 | 0 | 0 | 30 | T |
| *Characidium* sp. 1 | 3 | 14 | 0 | 0 | 0 | 0 | 17 | T |
| *Characidium* sp. 2 | 49 | 0 | 0 | 0 | 0 | 0 | 49 | T |
| Family Gasteropelecidae |  |  |  |  |  |  |  |  |
| *Gasteropelecus maculatus* | 6 | 0 | 0 | 0 | 0 | 0 | 6 | Po |
| Family Characidae |  |  |  |  |  |  |  |  |
| *Argopleura diquensis* | 4 | 10 | 0 | 0 | 0 | 0 | 14 | Pe |
| *Argopleura magdalenensis* | 98 | 224 | 0 | 0 | 0 | 0 | 322 | Pe |
| *Astyanax fasciatus* | 65 | 0 | 0 | 0 | 0 | 0 | 65 | Pe |
| *Astyanax filiferus* | 11 | 0 | 0 | 0 | 0 | 0 | 11 | Pe |
| *Astyanax gisleni* | 0 | 0 | 1 | 21 | 24 | 0 | 46 | Pe |
| *Astyanax magdalenae* | 54 | 0 | 0 | 0 | 0 | 0 | 54 | Pe |
| *Astyanax* sp*.* | 6 | 0 | 0 | 0 | 0 | 0 | 6 | Pe |
| *Bryconamericus caucanus* | 0 | 17 | 14 | 0 | 18 | 5 | 54 | Po |
| *Bryconamericus huilae* | 10 | 12 | 9 | 83 | 15 | 27 | 156 | Po |
| *Bryconamericus tolimae* | 0 | 100 | 12 | 50 | 0 | 0 | 162 | Po |
| *Bryconamericus* sp. 1 | 24 | 0 | 0 | 0 | 0 | 0 | 24 | Pe |
| *Bryconamericus* sp. 2 | 0 | 6 | 11 | 0 | 0 | 5 | 22 | Pe |
| *Bryconamericus* sp. 3 | 64 | 44 | 92 | 9 | 21 | 55 | 285 | Pe |
| *Creagrutus affinis* | 76 | 0 | 0 | 0 | 0 | 0 | 76 | Po |
| *Creagrutus brevipinnis* | 14 | 99 | 0 | 164 | 0 | 0 | 277 | Po |
| *Creagrutus guanes* | 0 | 163 | 0 | 0 | 0 | 0 | 163 | Po |
| *Creagrutus magdalenae* | 70 | 261 | 0 | 0 | 0 | 0 | 331 | Po |
| *Creagrutus* sp. 1 | 0 | 110 | 0 | 0 | 0 | 0 | 110 | Po |
| *Creagrutus* sp. 2 | 0 | 42 | 37 | 0 | 0 | 0 | 79 | Po |
| *Gephyrocharax melanocheir* | 165 | 4 | 0 | 0 | 0 | 0 | 169 | Pe |
| *Gephyrocharax* sp. | 0 | 0 | 0 | 7 | 0 | 0 | 7 | Pe |
| *Hemibrycon boquiae* | 64 | 49 | 20 | 174 | 1 | 5 | 313 | Pe |
| *Hemibrycon colombianus* | 0 | 0 | 17 | 0 | 0 | 0 | 17 | Pe |
| *Hemibrycon dentatus* | 20 | 149 | 0 | 8 | 0 | 0 | 177 | Pe |
| *Hemibrycon tolimae* | 0 | 5 | 74 | 0 | 0 | 0 | 79 | Pe |
| *Hyphessobrycon* sp. | 0 | 8 | 0 | 0 | 0 | 0 | 8 | Po |
| *Microgenys minuta* | 157 | 2 | 0 | 0 | 0 | 0 | 159 | Pe |
| *Roeboides dayi* | 40 | 0 | 0 | 0 | 0 | 0 | 40 | Po |
| *Saccoderma hastatus* | 25 | 47 | 0 | 0 | 0 | 0 | 72 | Pe |
| Family Bryconidae |  |  |  |  |  |  |  |  |
| *Brycon henni* | 0 | 14 | 9 | 3 | 44 | 1 | 71 | R |
| Family Erythrinidae |  |  |  |  |  |  |  |  |
| *Hoplias malabaricus* | 22 | 16 | 0 | 0 | 0 | 0 | 38 | Po |
| Family Ctenoluciidae |  |  |  |  |  |  |  |  |
| *Ctenolucius hujeta* | 9 | 0 | 0 | 0 | 0 | 0 | 9 | R |
|  |  |  |  |  |  |  |  |  |
| Order Siluriformes |  |  |  |  |  |  |  |  |
| Family Cetopsidae |  |  |  |  |  |  |  |  |
| *Cetopsis othonops* | 5 | 1 | 0 | 0 | 0 | 0 | 6 | Po |
| Family Trichomycteridae |  |  |  |  |  |  |  |  |
| *Trichomycterus banneaui* | 134 | 679 | 26 | 140 | 0 | 0 | 979 | T |
| *Trichomycterus caliensis* | 2 | 12 | 24 | 0 | 41 | 36 | 115 | T |
| *Trichomycterus latistriatus* | 0 | 3 | 5 | 0 | 0 | 1 | 9 | T |
| *Trichomycterus ruitoquensis* | 0 | 36 | 0 | 0 | 0 | 0 | 36 | T |
| *Trichomycterus stellatus* | 1 | 12 | 14 | 4 | 20 | 0 | 51 | T |
| *Trichomycterus striatus* | 19 | 227 | 68 | 8 | 0 | 0 | 322 | T |
| *Trichomycterus transandianum* | 0 | 1 | 7 | 0 | 0 | 0 | 8 | T |
| *Trichomycterus* sp. 1 | 0 | 3 | 32 | 0 | 0 | 0 | 35 | T |
| *Trichomycterus* sp. 2 | 0 | 0 | 0 | 55 | 0 | 0 | 55 | T |
| *Trichomycterus* sp. 3 | 0 | 0 | 0 | 0 | 0 | 8 | 8 | T |
| *Trichomycterus* sp. 4 | 74 | 0 | 0 | 0 | 0 | 0 | 74 | T |
| *Trichomycterus* sp. 5 | 0 | 264 | 0 | 0 | 0 | 0 | 264 | T |
| *Trichomycterus* sp. 6 | 0 | 1 | 0 | 0 | 0 | 0 | 1 | T |
| *Trichomycterus* sp. 7 | 0 | 0 | 0 | 16 | 0 | 0 | 16 | T |
| *Trichomycterus* sp. 8 | 0 | 0 | 0 | 6 | 0 | 0 | 6 | T |
| Family Astroblepidae |  |  |  |  |  |  |  |  |
| *Astroblepus chapmani* | 1 | 0 | 16 | 59 | 0 | 377 | 453 | T |
| *Astroblepus chotae* | 0 | 8 | 66 | 9 | 32 | 2 | 117 | T |
| *Astroblepus cirratus* | 2 | 5 | 4 | 3 | 1 | 131 | 146 | T |
| *Astroblepus cyclopus* | 0 | 4 | 5 | 35 | 0 | 1 | 45 | T |
| *Astroblepus fissidens* | 0 | 0 | 0 | 13 | 3 | 0 | 16 | T |
| *Astroblepus grixalvii* | 9 | 13 | 10 | 1 | 0 | 1 | 34 | T |
| *Astroblepus homodon* | 22 | 144 | 76 | 8 | 5 | 3 | 258 | T |
| *Astroblepus longifilis* | 0 | 39 | 66 | 14 | 64 | 6 | 189 | T |
| *Astroblepus micrescens* | 0 | 1 | 3 | 4 | 14 | 127 | 149 | T |
| *Astroblepus santanderensis* | 0 | 2 | 0 | 0 | 0 | 0 | 2 | T |
| *Astroblepus trifasciatus* | 0 | 0 | 11 | 0 | 14 | 1 | 26 | T |
| *Astroblepus unifasciatus* | 0 | 0 | 1 | 2 | 1 | 1 | 5 | T |
| *Astroblepus* sp. 1 | 0 | 0 | 5 | 1 | 5 | 5 | 16 | T |
| *Astroblepus* sp. 2 | 11 | 0 | 0 | 0 | 0 | 0 | 11 | T |
| *Astroblepus* sp. 3 | 0 | 0 | 0 | 0 | 0 | 21 | 21 | T |
| *Astroblepus* sp. 4 | 0 | 0 | 0 | 23 | 0 | 0 | 23 | T |
| *Astroblepus* sp. 5 | 0 | 0 | 0 | 0 | 0 | 48 | 48 | T |
| *Astroblepus* sp. 6 | 0 | 0 | 0 | 2 | 0 | 0 | 2 | T |
| *Astroblepus* sp. 7 | 0 | 0 | 0 | 0 | 0 | 17 | 17 | T |
| *Astroblepus* sp. 8 | 0 | 0 | 0 | 7 | 0 | 0 | 7 | T |
| *Astroblepus* sp. 9 | 0 | 0 | 0 | 21 | 0 | 0 | 21 | T |
| *Astroblepus* sp. 10 | 0 | 0 | 0 | 22 | 0 | 0 | 22 | T |
| *Astroblepus* sp. 11 | 0 | 0 | 0 | 0 | 27 | 0 | 27 | T |
| *Astroblepus* sp. 12 | 0 | 0 | 0 | 6 | 0 | 0 | 6 | T |
| *Astroblepus* sp. 13 | 0 | 0 | 0 | 1 | 0 | 0 | 1 | T |
| *Astroblepus* sp. 14 | 0 | 2 | 0 | 0 | 0 | 0 | 2 | T |
| *Astroblepus* sp. 15 | 0 | 0 | 15 | 0 | 0 | 0 | 15 | T |
| *Astroblepus* sp. 16 | 0 | 2 | 10 | 70 | 0 | 0 | 82 | T |
| *Astroblepus* sp. 17 | 11 | 0 | 0 | 0 | 0 | 0 | 11 | T |
| *Astroblepus* sp. 18 | 0 | 0 | 0 | 0 | 0 | 54 | 54 | T |
| *Astroblepus* sp. 19 | 0 | 0 | 0 | 0 | 0 | 2 | 2 | T |
| Family Loricariidae |  |  |  |  |  |  |  |  |
| *Ancistrus centrolepis* | 0 | 5 | 10 | 0 | 0 | 0 | 15 | Po |
| *Ancistrus* sp. | 0 | 1 | 37 | 0 | 0 | 0 | 38 | Po |
| *Chaetostoma fischeri* | 51 | 90 | 0 | 0 | 0 | 0 | 141 | T |
| *Chaetostoma leucomelas* | 52 | 250 | 26 | 0 | 0 | 0 | 328 | T |
| *Chaetostoma milesi* | 33 | 28 | 4 | 0 | 0 | 0 | 65 | T |
| *Chaetostoma thomsoni* | 51 | 384 | 0 | 0 | 0 | 0 | 435 | T |
| *Chaetostoma* sp. 1 | 15 | 2 | 0 | 0 | 0 | 0 | 17 | T |
| *Chaetostoma* sp. 2 | 52 | 65 | 20 | 0 | 0 | 0 | 137 | T |
| *Cordylancistrus* sp. 1 | 0 | 1 | 0 | 0 | 0 | 0 | 1 | T |
| *Cordylancistrus* sp. 2 | 2 | 8 | 0 | 0 | 12 | 0 | 22 | T |
| *Crossoloricaria variegata* | 1 | 0 | 0 | 0 | 0 | 0 | 1 | N-t b |
| *Dasyloricaria filamentosa* | 1 | 0 | 0 | 0 | 0 | 0 | 1 | N-t b |
| *Dolichancistrus carnegiei* | 0 | 0 | 0 | 0 | 0 | 64 | 64 | T |
| *Dolichancistrus* sp. | 0 | 5 | 49 | 0 | 0 | 0 | 54 | T |
| *Hypostomus hondae* | 12 | 3 | 0 | 0 | 0 | 0 | 15 | N-t b |
| *Lasiancistrus caucanus* | 210 | 85 | 59 | 0 | 0 | 0 | 354 | T |
| *Pterygoplichthys undecimalis* | 1 | 0 | 0 | 0 | 0 | 0 | 1 | N-t b |
| *Spatuloricaria gymnogaster* | 2 | 0 | 0 | 0 | 0 | 0 | 2 | N-t b |
| *Sturisoma aureum* | 34 | 0 | 0 | 0 | 0 | 0 | 34 | N-t b |
| *Sturisomatichthys leightoni* | 34 | 12 | 0 | 0 | 0 | 0 | 46 | N-t b |
| Family Heptapteridae |  |  |  |  |  |  |  |  |
| *Cetopsorhamdia boquillae* | 0 | 3 | 0 | 0 | 0 | 0 | 3 | T |
| *Cetopsorhamdia molinae* | 5 | 1 | 0 | 0 | 0 | 0 | 6 | T |
| *Cetopsorhamdia nasus* | 11 | 0 | 0 | 0 | 0 | 0 | 11 | T |
| Heptapteridae sp. * | 2 | 2 | 0 | 0 | 0 | 0 | 4 | Po |
| *Heptapterus* sp. | 21 | 42 | 0 | 0 | 0 | 0 | 63 | Po |
| *Imparfinis nemacheir* | 10 | 4 | 0 | 0 | 0 | 0 | 14 | T |
| *Imparfinis timana* | 0 | 0 | 0 | 56 | 0 | 0 | 56 | T |
| *Imparfinis usmai* | 0 | 1 | 0 | 0 | 0 | 0 | 1 | T |
| *Pimelodella chagresi* | 68 | 91 | 0 | 3 | 0 | 0 | 162 | Po |
| *Rhamdia quelen* | 29 | 40 | 0 | 2 | 0 | 0 | 71 | Po |
| Family Auchenipteridae |  |  |  |  |  |  |  |  |
| *Ageneiosus pardalis* | 17 | 0 | 0 | 0 | 0 | 0 | 17 | R |
| Family Pimelodidae |  |  |  |  |  |  |  |  |
| *Pimelodus blochii* | 7 | 1 | 0 | 0 | 0 | 0 | 8 | R |
| *Pimelodus grosskopfii* | 1 | 0 | 0 | 0 | 0 | 0 | 1 | R |
|  |  |  |  |  |  |  |  |  |
| Order Gymnotiformes |  |  |  |  |  |  |  |  |
| Family Apteronotidae |  |  |  |  |  |  |  |  |
| *Apteronotus eschmeyeri* | 3 | 0 | 0 | 0 | 0 | 0 | 3 | Pe |
| *Apteronotus rostratus* | 1 | 0 | 0 | 0 | 0 | 0 | 1 | Pe |
| Family Sternopygidae |  |  |  |  |  |  |  |  |
| *Sternopygus aequilabiatus* | 18 | 0 | 0 | 0 | 0 | 0 | 18 | Pe |
|  |  |  |  |  |  |  |  |  |
| Order Cyprinodontiformes |  |  |  |  |  |  |  |  |
| Family Rivulidae |  |  |  |  |  |  |  |  |
| *Cynodonichthys magdalenae* | 8 | 2 | 34 | 0 | 0 | 1 | 45 | Po |
| *Cynodonichthys* sp. | 3 | 0 | 0 | 0 | 0 | 0 | 3 | Po |
| Family Poeciliidae |  |  |  |  |  |  |  |  |
| *Poecilia caucana* | 13 | 115 | 54 | 0 | 6 | 0 | 188 | Po |
| *Poecilia reticulata* | 4 | 36 | 64 | 40 | 0 | 0 | 144 | Po |
| *Poecilia sphenops* | 42 | 0 | 0 | 0 | 0 | 0 | 42 | Po |
|  |  |  |  |  |  |  |  |  |
| Order Synbranchiformes |  |  |  |  |  |  |  |  |
| Family Synbranchidae |  |  |  |  |  |  |  |  |
| *Synbranchus marmoratus* | 3 | 1 | 4 | 0 | 0 | 0 | 8 | Po |
|  |  |  |  |  |  |  |  |  |
| Order Perciformes |  |  |  |  |  |  |  |  |
| Family Cichlidae |  |  |  |  |  |  |  |  |
| *Andinoacara latifrons* | 27 | 18 | 0 | 0 | 0 | 0 | 45 | Po |
| *Caquetaia kraussii* | 3 | 0 | 0 | 0 | 0 | 0 | 3 | Po |
| *Caquetaia umbrifera* | 12 | 0 | 0 | 0 | 0 | 0 | 12 | Po |
| *Geophagus steindachneri* | 90 | 29 | 13 | 0 | 0 | 117 | 249 | Po |
|  |  |  |  |  |  |  |  |  |
| Total | 2356 | 4217 | 1150 | 1163 | 368 | 1122 | 10376 |  |

* Possible new genus

Appendix 5. Diversity values of order zero (^0^*D*, species richness) for each regional elevation band. Assemblages with morphospecies are black circles and those with no morphospecies are white circles.
